# Functional Profile of CD8^+^ T-Cells in Response to HLA-A*02:01-Restricted Mutated Epitopes Derived from the Gag Protein of Circulating HIV-1 Strains from Medellín, Colombia

**DOI:** 10.3389/fimmu.2022.793982

**Published:** 2022-03-22

**Authors:** Alexandra Sánchez-Martínez, Liliana Acevedo-Sáenz, Juan Carlos Alzate-Ángel, Cristian M. Álvarez, Fanny Guzmán, Tanya Roman, Silvio Urcuqui-Inchima, Walter D. Cardona-Maya, Paula Andrea Velilla

**Affiliations:** ^1^ Grupo Inmunovirología, Departamento de Microbiología y Parasitología, Facultad de Medicina, Universidad de Antioquia UdeA, Medellín, Colombia; ^2^ Grupo Cuidado Enfermería CES, Facultad de Enfermería, Universidad CES, Medellín, Colombia; ^3^ Unidad de Micología Médica y Experimental, Corporación para Investigaciones Biológicas, Medellín, Universidad de Santander (CIB-UDES), Bucaramanga, Colombia; ^4^ Grupo de Inmunología Celular e Inmunogenética, Facultad de Medicina, Universidad de Antioquia UdeA, Medellín, Colombia; ^5^ Núcleo de Biotecnología Curauma, Pontificia Universidad Católica de Valparaíso, Valparaíso, Chile; ^6^ Grupo Reproducción, Facultad de Medicina, Universidad de Antioquia UdeA, Medellín, Colombia

**Keywords:** CD8^+^ T-cells, T-cell epitopes, HIV-1, HLA-A*02, polyfunctionality, class I HLA-peptide binding affinity, gag-derived peptides

## Abstract

CD8^+^ T-cells play a crucial role in the control of HIV replication. HIV-specific CD8^+^ T-cell responses rapidly expand since the acute phase of the infection, and it has been observed that HIV controllers harbor CD8^+^ T-cells with potent anti-HIV capacity. The development of CD8^+^ T-cell-based vaccine against HIV-1 has focused on searching for immunodominant epitopes. However, the strong immune pressure of CD8^+^ T-cells causes the selection of viral variants with mutations in immunodominant epitopes. Since HIV-1 mutations are selected under the context of a specific HLA-I, the circulation of viral variants with these mutations is highly predictable based on the most prevalent HLA-I within a population. We previously demonstrated the adaptation of circulating strains of HIV-1 to the HLA-A*02 molecule by identifying mutations under positive selection located in GC9 and SL9 epitopes derived from the Gag protein. Also, we used an *in silico* prediction approach and evaluated whether the mutations found had a higher or lower affinity to the HLA-A*02. Although this strategy allowed predicting the interaction between mutated peptides and HLA-I, the functional response of CD8^+^ T-cells that these peptides induce is unknown. In the present work, peripheral blood mononuclear cells from 12 HIV-1^+^ HLA-A*02:01^+^ individuals were stimulated with the mutated and wild-type peptides derived from the GC9 and SL9 epitopes. The functional profile of CD8^+^ T-cells was evaluated using flow cytometry, and the frequency of subpopulations was determined according to their number of functions and the polyfunctionality index. The results suggest that the quality of the response (polyfunctionality) could be associated with the binding affinity of the peptide to the HLA molecule, and the functional profile of specific CD8^+^ T-cells to mutated epitopes in individuals under cART is maintained.

## Introduction

HIV-1/AIDS still represents one of the most significant health issues worldwide (1). Since the beginning of the pandemic, approximately four decades ago, no treatment to eradicate the virus or an effective prophylactic vaccine has been developed (2). Despite the success of combined antiretroviral therapy (cART) in reducing AIDS-associated deaths and changed the prospects of HIV/AIDS disease, this therapy has pitfalls such, the non-adherence of the patients, poor tolerability, drug resistance, and drug interactions (3), but also the continuous HIV replication in compartments and persistence of viral reservoirs (4). These problems point out the need for further therapeutic strategies that contribute to HIV control. Recently, alternative therapeutic strategies against HIV-1 have focused on inducing specific CD8^+^ T-cell responses that are crucial in controlling HIV-1 replication (5). HIV-specific CD8^+^ T-cell responses rapidly expand since the acute phase of the infection, and their direct effector function can be observed throughout the chronic phase of the disease (6). Furthermore, it has been observed that individuals that maintain low to undetectable viral loads in the absence of cART for at least one year, known as HIV-1 controllers, harbor CD8^+^ T-cells with a potent anti-HIV-1 ability (5, 7). Thus, a vaccine targeted to induce a strong anti-HIV CD8^+^ T-cell response would be a promising therapeutic approach.

Efforts in developing CD8^+^ T-cell-based vaccine against HIV-1 have been focused on the search of immunodominant epitopes circulating in a population (8), and the identification of high affinity epitopes that bind strongly to the respective HLA-I molecule and induce a potent CD8^+^ T-cell response (9). However, the strong immune pressure of CD8^+^ T-cells causes the selection of viral variants with mutations in immunodominant epitopes (10). The response to such mutated epitopes has been evaluated in different studies; for example, the R3K substitution in the wild-type LARNCRAP (LR9) epitope selected for the HLA-A*03:01 allele, induces a greater magnitude of response in CD8^+^ T-cells compared to the WT epitope (11). Also, Karlsson et al. reported CD8^+^ T-cell response to the E93D mutant variant of the HLA-B*08-restricted EVKDTKEAL epitope and the effectiveness of the CD8^+^T cell response in killing cells infected with this viral variant (12). In addition, the M41L mutation, selected in the presence of didanosine and stavudine, localizes to the ALVAICTEM epitope presented by HLA-A*02/*03 molecules, is associated with increased epitope immunogenicity *in vivo* and CD8+ LT activation and expansion *in vitro* (13). Since HIV-1 mutations are selected under the context of a specific HLA-I, the circulation of viral variants with these mutations is highly predictable based on the most prevalent HLA-I within a population (14), representing one of the essential factors for HIV-1 diversification at the population level.

A previous study from our group demonstrated the adaptation of circulating HIV-1 strains from Medellín, Colombia, to the most prevalent HLA-I alleles in the population, HLA-A*02:01. Different mutations mapped into HLA-I-specific GC9 and SL9 epitopes associated with decreased or increased binding affinity towards HLA-A*02:01 were identified (15). Interestingly, our analyses showed that two different mutations in the HLA-A*02-restricted GC9 epitope, S54A, and S54T, significantly increased the epitope binding affinity towards HLA-A*02; also showed a mutation, Y79F/T84V/L85F, associated with low HLA-binding affinity in HLA-A*02-restricted SL9, which has been considered immunodominant epitope (16). However, the experimental evaluation of the peptide immunogenicity is required to validate HLA-I ligands prediction, since it has been shown that the binding affinity of the epitope to HLA-I is not the only determinant variable (17). Therefore, we evaluated the functional profile of the CD8^+^ T-cell response to positively selected mutations located in HLA-A*02:01-restricted epitopes from HIV strains that circulate in our population.

## Materials and Methods

### Study Design and Sample Collection

The Bioethics Committee from the Institute of Medical Research, School of Medicine, Universidad de Antioquia, approved this study design and informed consent. This was an observational and descriptive study, divided into two phases. We first identified individuals who met the inclusion criteria and expressed the HLA-A*02 allele. Then, the sample size was determined taking into account the allelic frequency of HLA-A*02 in Colombia (20%), and the number of HIV-1 infected individuals who attend the HIV healthcare program of “Corporación para Investigaciones Biológicas – CIB”, with a confidence level of 95% and a precision of 10%, using the software Epidat 4.2. The inclusion criteria were: i) Residency in Medellín, Colombia; ii) Confirmed HIV-1 infection; iii) Antiretroviral therapy naïve or on therapy for less than five years. Exclusion criteria were: i) Less than 18 years of age; ii) Active opportunistic infection; iii) Received chemotherapy/immunosuppressive treatment. In the second phase of the study, once the study population was identified, the functional profile of the CD8^+^ T-cells response was evaluated.

### Measurement of T-cell Counts and HLA-I Typing

CD4^+^ and CD8^+^ T-cell counts were performed in peripheral blood (PB) using flow cytometry. Briefly, 100 μL of PB was incubated with conjugated human anti-CD3, anti-CD4, and anti-CD8 monoclonal antibodies; red blood cells were lysed using a commercial cell lysis buffer (BD Biosciences) and washed twice with phosphate buffer saline (PBS) (Sigma-Aldrich, St. Louis, MO). The cells were acquired on the BD LSRFortessa flow cytometer with FACSDiva software v 8.0.1 (BD Biosciences). HLA-I typing was performed with genomic DNA extracted from PB through a phenol/chloroform DNA extraction protocol. In addition, HLA-A typing was performed by the sequence-specific oligonucleotide (SSO) technology, using the Lifecodes^®^ HLA-SSO Typing Kit (Immucor Transplant Diagnostics, Inc., Stanford, CT) and measured using the Luminex^®^100/200TMinstrument (Luminex, Austin, TX, USA), as was previously reported (15).

### Peptide Synthesis and Purification

Peptide synthesis was performed according to the Fmoc/tBu standard strategy using a “tea-bag” protocol (18). Peptides were characterized by high-performance liquid chromatography (HPLC) in a JASCO system (JASCO Corp., Tokyo, Japan), and molecular mass was determined by electrospray-mass spectrometry (ESI–MS) in an LCMS-2020 ESI–MS equipment (Shimadzu Corp., Kyoto, Japan). Peptides were purified using preparative Clean-Up^®^ CEC18153 C-18 columns (UCT. Bristol. PA. USA) and eluted with an acetonitrile/water gradient from 10 to 60% (v/v). Fractions were analyzed by HPLC and ESI–MS. Afterward, peptides were lyophilized, reconstituted in sterile water (1 mg/mL), and stored at −20°C.

### *In vitro* Stimulation of CD8^+^ T-cells

Peripheral blood mononuclear cells (PBMCs) were isolated through density gradient (Lymphoprep, STEMCELL Technologies Inc., Vancouver, Canada*)* by centrifugation at 400 x g for 30 minutes and cultured in 96-well V bottom plates (Costar, Corning, NY) with a density of 4 x 10^6^ cells/mL in RPMI-1640 medium supplemented with 10% fetal bovine serum (FBS), 100 U/mL of penicillin, 100 μg/mL of streptomycin and 2 mM L-glutamine (complete medium; all from Gibco, Carlsbad, CA). PBMCs were stimulated with 10 μg/mL of each peptide (**Table 1**), in presence of 1 μg/mL of both anti-CD28 (clone: CD28.2, eBioscience) and anti-CD49d (clone: 9F10, eBioscience). Cells stimulated only with anti-CD28 and anti-CD49d antibodies were used as the negative control. The PBMCs were stimulated with 1 μg/mL of staphylococcal enterotoxin B (SEB) from *Staphylococcus aureus* (Sigma-Aldrich) or with 50 ng/mL of phorbol 12-myristate 13-acetate (PMA) and 500 ng/mL of ionomycin (both from Sigma-Aldrich) was also included in the analysis. All stimulated cells were incubated for 12 h at 37˚C in 5% CO_2_, in the presence of 10 μg/mL of brefeldin A and monensin (both from Thermo Fisher), as well as anti-human CD107a (clone H4A3, BD).

**Table 1:** List of the wild type and mutated GC9 and SL9 epitopes.

Protein/	Position in Gag	Epitope	Wild type sequence	Mutated sequence	Abbreviation mutated sequence	Peptide affinity relative to WT
Gag-p17	49-57	GC9	GLLETSEGC	GLLETAGGC	S54A/E55G	Decrease
				GLLETAEGC	S54A	Increase
				GLLETTEGC	S54T	Increase
Gag-p17	77-85	SL9	SLYNTVATL	SLFNTVAVF	Y79F/T84V/L85F	Decrease

### Intracellular Cytokine Staining

After PBMCs stimulation, cells were washed with PBS and incubated at 4°C for 30 minutes with Fixable Viability Dye eFluor 506 (eBioscience), and with the anti-CD3-AF700 (clone: UCHT1, eBioscience) and anti-CD8-eFluor450 (clone: RPA-T8, eBiocience) antibodies. Next, cells were fixed and permeabilized with Foxp3 Fixation/Permeabilization Buffer (eBioscience) and then incubated with the following conjugated antibodies: anti-IL-2- (clone: 5344.111, BD), anti-Granzyme B (clone: GB11, BD), anti-Perforin (clone: B-D48, Biolegend), anti-IFN-γ (clone: 4S.B3, Biolegend), anti-TNF-α (clone: MAb11, eBioscience) and anti-IL-10 (clone: JES3-9D3, eBioscience). Cells were acquired on an LSR Fortessa flow cytometer using the BD FACSDiva software v 8.0.1 (BD). At least 100,000 CD3^+^ events were recorded. Fluorescence minus one (FMO) control for the effector molecules was included to define positive thresholds. For fluorochrome spillover compensation, unstained and single-stained cells were used with each of the fluorochrome-labeled antibodies. Then, automatic compensation was performed on LSR Fortessa.

### Analysis of Flow Cytometry Data

FlowJo Software version 10.4 (Tree Star, Inc, Ashland, OR, USA) was used to analyze flow cytometry data. Cells were analyzed following the gating strategy shown in **Supplementary Figure 1**. Boolean gating was performed to create a full array of possible combinations of expression of effector molecules, with up to 64 response patterns from the CD8^+^ T-cell gate. Data were reported after background subtraction (from the negative control) and background correction. The background correction was defined according to the number of functions (19). A value equal or higher than 0.05%, 0.005%, and 0.0005% were considered positive for cells showing 1-2, 3-4, and 5-6 functions, respectively. Data were visually represented using SPICE software v5.35 (Vaccine Research Center, NIAID/NIH, Bethesda, MD, USA). Also, the polyfunctional index (PI) was determined, which numerically evaluates the degree and variation of polyfunctionality (20), reducing the n-dimensional polyfunctionality profiles to a one-dimensional index value, using the mathematical equation: $\text{Polyfunctionality index}=\Sigma_{i=0}^{n}\;F_{i}.\;{\left(\frac{i}{n}\right)}^{q},100=\Sigma_{i=0}^{n}\;F_{i}$ , and *F_i_ ≥* 0 for all i, where n > 0 is the number of cell functions, *F_i_* is the frequency (%) of cells performing *i* functions and *q ≥* 0 is the parameter that modulates the differential weight assignment of each *F_i_*. The algorithm requires that the sum of all *F_i_* equals 100 and that all *F_i_* are absolute values (20), through the FunkyCells ToolBox software (www.FunkyCells.com).

### Statistical Analysis

GraphPad Prism software v. 7.0 (GraphPad Software, La Jolla, CA) was used for statistical analysis. Non-parametric analyses were performed. Data were presented as median and interquartile range. The Wilcoxon signed-rank test was used for the comparison of paired data. In all cases, a P-value <0.05 was considered significant.

## Results

### Demographic and Clinical Characteristics of the Study Population

Twelve HIV-1 infected individuals were identified with the HLA-A*02:01 allele; in this study population, 75% were men with a median age of 45 years, which is consistent with the reported behavior of the infection in Colombia (21). The median time of diagnosis was 50.5 months. In most individuals, the CD4^+^ T-cell count was above 400 cells/μL, with a median of 527 cells/μL and a CD4^+^/CD8^+^ T-cell ratio of 0.8. Nine out of 12 (75%) individuals were under cART, with a median time on therapy of 46 months, all of them with undetectable viral load (**Table 2**). In general, there is no evidence of a marked immunological deterioration in these individuals, which may be associated with rapid initiation of antiretroviral therapy after the diagnosis of the infection (median of 2 months).

**Table 2:** Clinical characteristics of the study cohort.

Parameter	Value
Age, years, median (IQR)	45 (30.8-51)
Gender, Female/Male	3/9
CD4^+^ T-cell counts, cells/μL, median (IQR)	527(423-591)
Percentage CD4^+^ T-cells among CD3^+^ T-cells, median (IQR)	26.1(23-30.1)
CD4^+^/CD8^+^ T-cell ratio, median (IQR)	0.8 (0.6-1.6)
Time since diagnosis, months, median (IQR)	50.5 (43-57)
% receiving cART	75
Time on cART, months, median (IQR)	46 (31-54)
Time between diagnosis and cART initiation, months, median (IQR)	2 (1-15)
Viral load at the time of cART initiation, copies/mL, median (IQR)	25,148 (10,525.2-84,485)

IQR, interquartile range; cART, combination antiretroviral therapy.

### CD8^+^ T-cell Response to Polyclonal Antigen-Independent Stimulation Is Maintained in the Study Population

**Figure 1:**
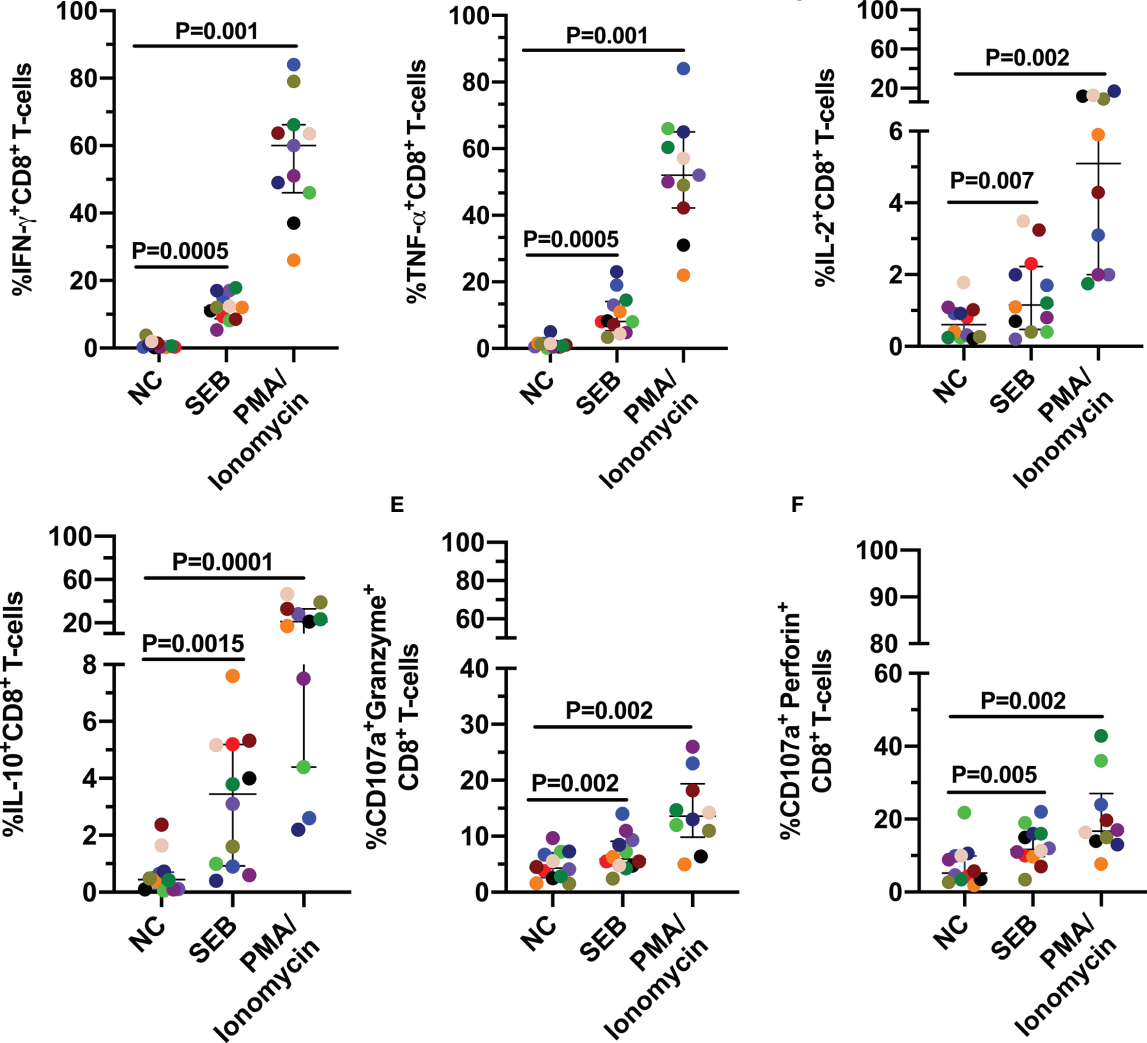
CD8^+^ T-cell response to polyclonal antigen-independent stimulation. Frequencies of CD8^+^ T-cells positive for IFN-γ **(A)**, TNF-α **(B)**, IL-2 **(C)**, IL-10 **(D)**, and CD107a together with granzyme B **(E)** or perforin **(F)**. Each point represents the CD8^+^ T-cell response in the negative control (NC), and after stimulation with SEB and PMA/Ionomycin (PMA) for 12 hours. The Wilcoxon test was used to compare the response of cells stimulated with each polyclonal stimulus and NC. Plots depict median and IQR, n = 10-12.

Considering that previous reports have shown alteration in the response of CD8^+^ T-cells to different stimuli, even upon prolonged cART (22), we initially focused our analysis on the expression of IFN-γ, TNF-α, IL-2, IL-10 cytokines and as well as in cytotoxic molecules like granzyme B and perforin in CD8^+^ T-cells after oligoclonal and pharmacological stimulation with SEB or PMA/Ionomycin respectively. In addition, the surface expression of CD107a was also evaluated as a marker of cell degranulation (23). As shown in **Figure 1**, all patients exhibited CD8^+^ T-cell responses to both polyclonal stimuli. Furthermore, IFN-γ and TNF-α were the most highly expressed cytokines (**Figures 1A, B**), whereas IL-2 and IL-10 were produced to the lowest extent (**Figures 1C, D**). Also, *de novo* expression of granzyme B and perforin was observed, evaluated by their coexpression with CD107a molecule, in response to both stimuli (**Figures 1E, F**).

**Figure 2:**
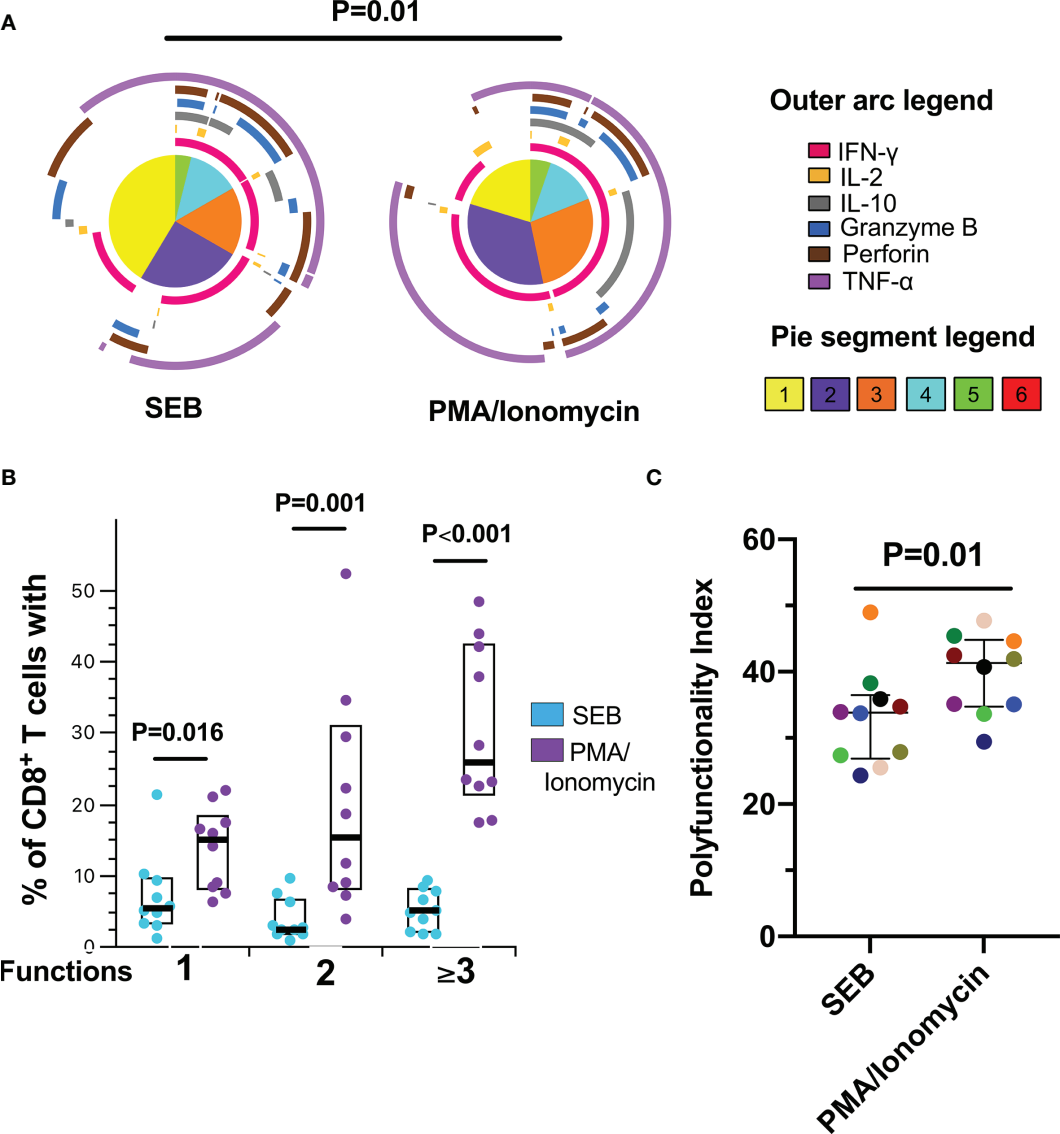
Polyfunctional CD8^+^ T-cell response to polyclonal antigen-independent stimulation. **(A)** Pie charts represent the average frequencies of CD8^+^ T-cells cells secreting cytokines and cytotoxic molecules producing every possible combination of the six molecules analyzed after stimulation with Staphylococcal Enterotoxin B (SEB) and PMA/Ionomycin (PMA) for 12 hours SEB and PMA/Ionomycin. The segments within the pie chart denote populations producing different combinations of molecules (pie segment legend). The size of the pie segment correlates to the frequency of the particular population. The arcs around the circumference indicate the particular molecule (see outer arc legend) produced by the proportion of cells that lie under the arc. Parts of the pie surrounded by multiple arcs represent polyfunctional cells. **(B)** Percentage of cells exhibiting one, two, or more than three functions. **(C)** Polyfunctionality index. Wilcoxon test was used for matched-paired samples. Plots depict the median and IQR, n = 10.

To characterize the functional profile of the CD8^+^ T-cells response after polyclonal antigen-independent stimulation, a Boolean gating was performed to create a full array of possible combinations of the expression of effector molecules. Consistent with a more potent activating signal (24), CD8^+^ T-cells were more polyfunctional in response to PMA/Ionomycin than SEB (**Figures 2A, B**). In addition, the production of IFN-γ and TNF-α dominated the response to both polyclonal stimuli, together with the expression of granzyme B and perforin (**Figure 2A**). Accordingly, a higher polyfunctionality index (PI) in response to PMA/Ionomycin compared to SEB was observed (**Figure 2C**). Thus, despite the different magnitudes of the functional response among the individuals, these results indicate that CD8^+^ T-cells from this cohort of HIV-infected individuals exhibit lytic and non-lytic effector functions and polyfunctionality following polyclonal antigen-independent stimulation, which may be related to the degree of immune reconstitution observed in most of these individuals.

### Mutated Peptides Induce a Functional Response of CD8^+^ T-cells

**Figure 3:**
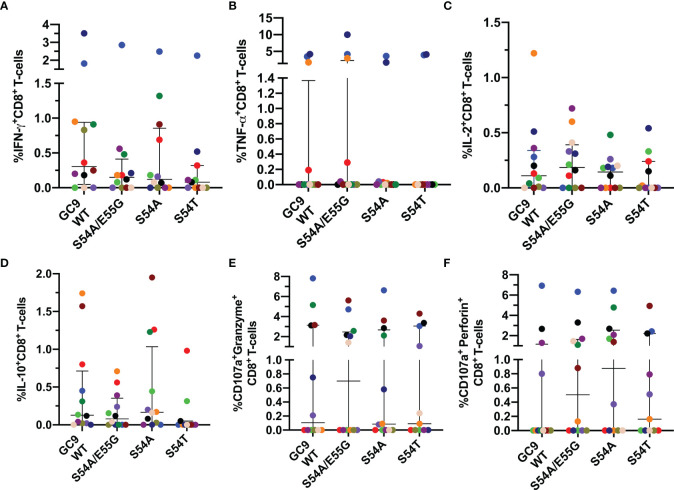
Cytokine mediated and cytotoxic CD8^+^ T-cell response to GC9 WT, S54A/E55G, S54A and S54T. Frequency of CD8^+^ T-cells positive for IFN-γ **(A)**, TNF-α **(B)**, IL-2 **(C)**, and IL-10 **(D)**, CD107a co-expressed granzyme B **(E)** or CD107a co-expressed perforin **(F)**. Each point represents the CD8^+^ T-cell response in an individual to each peptide (10 µg/mL) after subtraction of the negative control. Wilcoxon test was used for matched-paired samples and comparisons were made between each mutated peptide with the wild type version (GC9 WT). Plots depict the median and IQR, n = 11-12.

The response of CD8^+^ T-cells to the wild type (GC9 WT) and three mutated peptides of HIV-1 Gag protein (S54A/E55G, S54A, and S54T) located in the GC9 epitope (position 49-57) (**Table 1**) was evaluated. It was observed that most of the individuals responded to both WT and mutated peptides. There were no significant differences in the frequencies of IFN-γ-producing CD8^+^ T-cells between the GC9 WT and the mutated peptides S54A/E55G, S54A, and S54T (**Figure 3A**). In general, nine individuals showed a positive response to the WT peptide, and from them, seven, five, and five also showed response to S54A/E55G, S54A and S54T mutated peptides, respectively. However, a particular pattern was observed among the mutated peptides. Specifically, in four individuals, the IFN-γ response to the S54A peptide was stronger than GC9 WT, whereas, in three individuals in which no response to GC9 WT was observed, a low but detectable frequency of IFN-γ^+^ CD8^+^ T-cells was obtained in response to the S54A peptide (see light green point, **Figure 3A**). Also, one individual showed a high frequency of IFN-γ-producing CD8^+^ T-cells in response to S54A/E55G, S54A, and S54T peptides vs. GC9 WT peptide (**Figure 3A**, light blue point), whereas another individual responded only to WT peptide stimulation (**Figure 3A**, golden points). These results suggest that the S54A peptide, associated with high affinity to the HLA molecule, could be a better inducer of an IFN-γ CD8^+^ T-cell response than the other peptides.

Concerning TNF-α-production, in 3 out of 10 individuals was found a higher frequency of TNF-α-producing CD8^+^ T-cells in response to the GC9 WT peptide, where two of them also maintained a high response to the three mutated peptides (**Figure 3B**). About the IL-2 response, most individuals respond to both WT and mutated peptides, where one individual exhibited a higher frequency of IL-2^+^ CD8^+^ T-cells in response to the GC9 WT and S54A/E55G peptides (**Figure 3C**). Also, it was observed IL-10-expressing CD8^+^ T cells in response to WT and the mutated peptides S54A/E55G and S54A (**Figure 3D**). Noticeably, most individuals who responded with IFN-γ production also responded with IL-2 and IL-10 production.

Regarding the capacity of CD8^+^ T-cells to *de novo* cytotoxic molecules, it was observed that most individuals responded to mutated peptides (**Figure 3**); in some of them, with a higher frequency of CD107a^+^Perforin^+^ CD8^+^ T-cells compared to WT peptide (**Figure 3F**). Noticeably, the individual who showed the highest frequency of IFN-γ and TNF-α producing CD8^+^ T-cells in response to all GC9 peptide also had the most increased *de novo* production of granzyme B and perforin (**Figures 3E, F**, blue points). This individual was the only one homozygous for HLA-A*02 in our study cohort. Together, these results suggest that the mutated peptides induce a functional response of CD8^+^ T-cells, where all individuals showed at least one positive response to any of the cytokines evaluated.

### Higher Polyfunctionality Index in Response to GC9 S54T Peptide Is Associated With High HLA-A*02:01 Binding Affinity

**Figure 4:**
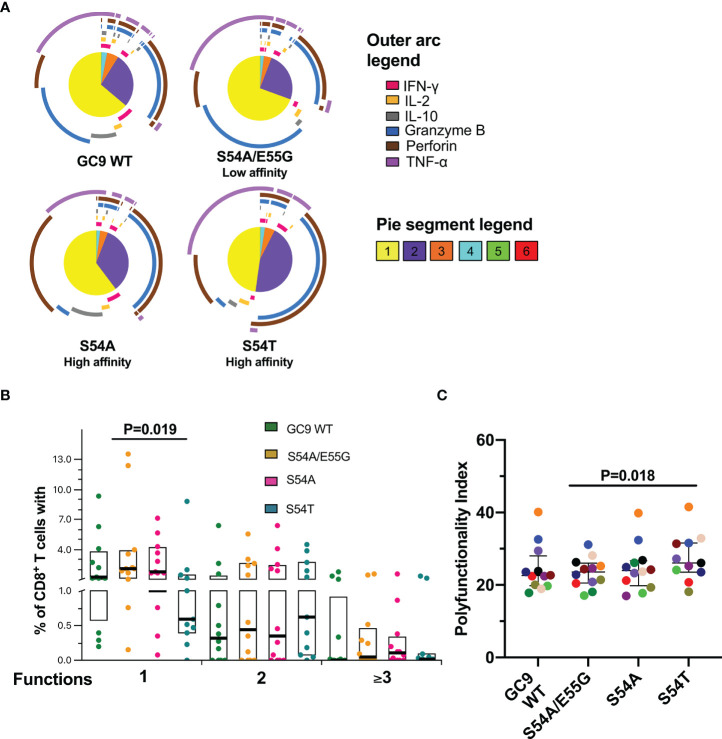
Functional profile of CD8^+^ T-cell response to GC9 WT, S54A/E55G, S54A and S54T. **(A)** Pie charts represent the average frequencies of CD8^+^ T-cells cells secreting cytokines and cytotoxic molecules producing every possible combination of the six molecules analyzed in response to GC9 WT and mutated peptides (10 µg/mL). The size of the pie segment correlates to the frequency of the particular population. The arcs around the circumference indicate the particular molecule (see outer arc legend) produced by the proportion of cells that lie under the arc. Parts of the pie surrounded by multiple arcs represent polyfunctional cells. **(B)** Percentage of cells responding through one, two, or more than three functions. **(C)** Polyfunctionality index. Wilcoxon test was used for matched-paired samples and comparisons were made between each mutated peptide with the wild type version (GC9 WT), but also among mutated variants. Plots depict the median and IQR, n = 11-12.

The functional quality of the CD8^+^ T-cell response was evaluated, considering that the polyfunctionality of these cells has been associated with the natural control of HIV-1 (25). As observed in **Figures 4A, B**, all the peptides from the GC9 epitope mostly induced a monofunctional response, being significantly lower the percentage of monofunctional cells after stimulation with the high HLA-A*02:01 binding affinity S54T peptide compared to the low binding affinity S54A/E55G peptide. Similar to GC9 WT peptide, TNF-α (purple arc) and granzyme B (blue arc) production dominated the monofunctional response to S54A/E55G peptide, while for the peptides associated with high affinity, most of the response was at the expense of TNF-α and perforin (brown arc), with a higher proportion of perforin-producing cells with S54A peptide (**Figure 4A**). The granzyme B^+^ perforin^+^ profile was dominant in cells exhibiting two functions, followed by the IFN-γ^+^ TNF-α^+^ profile. Cells performing three or more functions were mainly constituted by TNF-α^+^ granzyme B^+^ perforin^+^ cells **Figure 4A**). Consistent with the lower proportion of monofunctional cells, a higher index of polyfunctionality induced with peptide S54T was observed, being significant only compared to the S54A/E55G peptide (**Figure 4C**). Together, the results suggest that the quality of the functional response of CD8+ T-cells could be better in response to S54T peptide, associated with higher HLA binding affinity.

### Lower Frequency of CD8^+^ T-Cell Co-expressing CD107a^+^ Granzyme B^+^ Is Observed to the Low HLA-A*02:01 Binding Affinity Y79F/T84V/L85F Peptide

**Figure 5:**
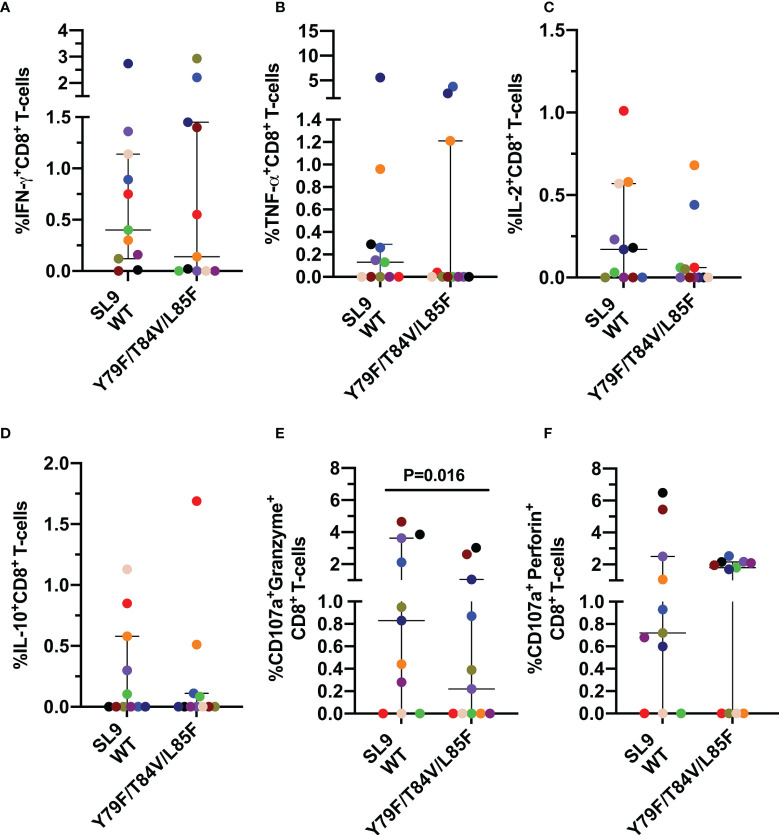
Cytokine mediated and cytotoxic CD8^+^ T-cell response to SL9 WT and Y79F/T84V/L85F. Frequency of CD8^+^ T-cells positive for IFN-γ **(A)**, TNF-α **(B)**, IL-2 **(C)**, IL-10 **(D)**, CD107a co-expressed granzyme B **(E)** or CD107a co-expressed perforin **(F)**. Each point represents the CD8^+^ T-cell response in an individual to each peptide (10 µg/mL) after subtraction of the negative control. Wilcoxon test was used for matched-paired samples. Plots depict the median and IQR, n = 11.

Also, we determined the pattern and magnitude of the CD8^+^ T-cell response to the SL9 WT peptide and its mutated form Y79F/T84V/L85F, associated with low affinity to the HLA-I molecule (**Table 1**). Similar to the GC9 epitope, a heterogeneous response was observed in the CD8^+^ T-cell stimulated with SL9 WT or Y79F/T84V/L85F peptides, with most individuals responding to the WT peptide (**Figures 5A–D**). No significant difference was observed for any of the cytokines evaluated. However, three individuals exhibited a higher frequency of IFN-γ^+^ CD8^+^ T-cells (**Figure 5A**), in response to the mutated peptide than SL9 WT. In contrast, two individuals showed a higher frequency of TNF-a or IL-2^+^ CD8^+^ T-cells (**Figures 5B, C**).

However, a lower frequency of CD107a^+^ granzyme B^+^ CD8^+^ T-cells in response to the mutated peptide was observed than the SL9 WT peptide (**Figure 5E**), suggesting that the lower HLA binding affinity of the Y79F/T84V/L85F peptide impairs the cytotoxic capacity of CD8^+^ T-cells. No difference was observed for CD107a^+^ perforin^+^ CD8^+^ T-cells (**Figure 5F**).

### Lower Polyfunctionality Index in Response to SL9 Y79F/T84V/L85F Peptide Is Associated With Lower HLA-A*02:01 Binding Affinity

**Figure 6:**
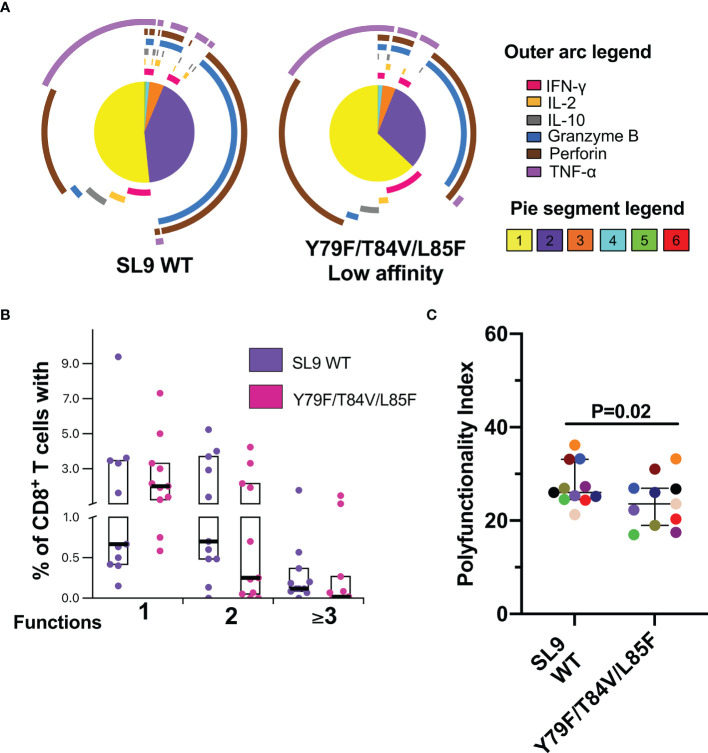
Polyfunctional CD8^+^ T-cell response to SL9 WT and Y79F/T84V/L85F. **(A)** Pie charts represent the average frequencies of CD8^+^ T-cells cells secreting cytokines and cytotoxic molecules producing every possible combination of the six molecules analyzed in response to SL9 WT and mutated peptides (10 µg/mL). The size of the pie segment correlates to the frequency of the particular population. The arcs around the circumference indicate the particular molecule (see outer arc legend) produced by the proportion of cells that lie under the arc. Parts of the pie surrounded by multiple arcs represent polyfunctional cells. **(B)** Percentage of cells responding through one, two, or more than three functions. **(C)** Polyfunctionality index. Wilcoxon test was used for comparison. Plots depict the median and IQR, n = 11.

Finally, the functional profile of the CD8^+^ T-cell response to the SL9 WT and Y79F/T84V/L85F was evaluated. Although no significant difference was found, the Y79F/T84V/L85F peptide exhibited a higher monofunctional response than SL9 WT (**Figures 6A, B**). Perforin expression dominated the monofunctional cell response for both types of peptides, and bifunctional responses were predominantly granzyme B^+^ perforin^+^ (**Figure 6A**). The responses of three functions were predominantly TNF-α^+^granzyme B^+^perforin^+^, and four functions were essentially IFN-γ^+^TNF-α^+^granzyme B^+^perforin^+^ (**Figure 6A**). Interestingly, the PI for Y79F/T84V/L85F was significantly lower than SL9 WT (P=0.02, **Figure 6C**), suggesting that the CD8^+^ T-cell response quality could be affected by this mutation.

### The Functional Profile Against Peptides Is Influenced by the Degree of Immunological Reconstitution and the Time of Treatment

**Figure 7:**
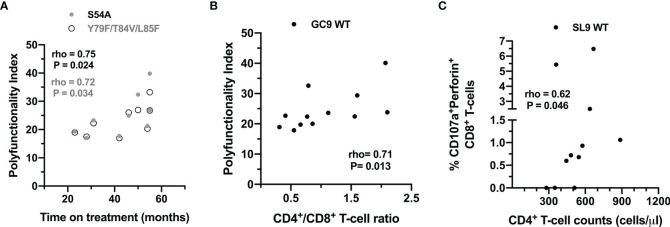
Association between clinical variables and the CD8^+^ T-cell response to wild type and mutated peptides. **(A)** Correlation between the PI of CD8^+^ T-cells after stimulation with the S54A (black dots) and Y79F/T84V/L85F peptides (gray dots), and the time on treatment of the HIV-1-infected individuals. **(B)** Correlation between the PI of CD8^+^ T-cells after stimulation with the GC9 WT and CD4:CD8 ratio. **(C)** Correlation between the frequency of CD107a^+^perforin^+^ CD8^+^ T-cells after stimulation with the SL9 WT and CD4^+^ T-cell counts. The rho and P-value of the Spearman test are shown.

Also, the association between clinical variables and the functional response of CD8^+^ T-cells to the peptides was evaluated. As shown in **Figure 7**, the functional profile against the WT peptides of the two epitopes is influenced by the degree of immunological reconstitution (CD4^+^ T-cell count and CD4^+^/CD8^+^ T-cell ratio); but also, the functional profile to the mutated peptides S54A and Y79F/T84V/L85F could be associated with the treatment time.

## Discussion

The induction of HIV-specific CD8^+^ T-cell responses is postulated as a promising strategy for developing an HIV vaccine and alternative immune therapy in HIV-infected individuals (26). The STEP vaccine trial assessed the efficacy of the MRK Ad5 HIV-1 gag/pol/nef vaccine, designed to stimulate a T-cell mediated immunity. The vaccine was shown to be highly effective in inducing CD8+ T cells in most of those vaccinated; however, it did not prevent HIV-1 infection or reduce early plasma viremia (27). Nonetheless, a follow-up analysis suggested that a higher number of Gag peptides showed increased control of HIV-1 replication of infected individuals who previously received the vaccine (28). Another example of a CD8^+^ T-cell-based vaccine is the HIVconsv immunogen, which consists of a chimeric protein assembled from highly conserved domains derived from HIV-1 genes Gag, Pol, Vif, and Env (29). This vaccine strategy focuses exclusively on eliciting T-cell responses toward more conserved and protective epitopes and has shown to induce high frequencies of polyfunctional and long-lived HIV-specific CD8^+^ T-cells capable of controlling HIV-1 replication *in vitro* (28–31). Moreover, this vaccine strategy has been evaluated in phase I clinical trial with a therapeutic purpose consisting of a cohort of HIV-1^+^ individuals under cART. A shift in immunodominance profiles of HIV-1-specific cells toward more conserved epitopes was observed (32). Although this vaccine strategy did not show a significant decrease in viral reservoir size, these data encourage further clinical development of this approach to a T-cell-based vaccine against HIV-1.

Identifying immunodominant peptides to activate CD8^+^ T-cells is a field of active research, pointing to the design of vaccines that elicit cellular immune responses (33). In this regard, peptide binding affinity to the HLA-I molecule is a crucial determinant of the epitopes to be considered as vaccine components (34). Previous studies postulated that the peptide binding affinity to the HLA-I molecule determines its immunogenicity (35, 36). Consistently, a study by Colleton et al. (37) observed that potential HIV-derived epitopes having a high binding affinity to its HLA-I molecule tend to be associated with a better immune response, an increase in the CD4^+^ T-cell counts, and a slow disease progression.

The most prevalent HLA-I allele is HLA-A*02, and alleles grouped into the HLA-A2 supertype are expressed in multiple populations around the world (38). For instance, in the Colombian population, the HLA-A*02 allele frequency is 22.5% (39). Alleles belonging to the HLA-A2 supertype are characterized by the ability to present an extensive repertoire of peptides with a strong binding affinity compared to other alleles, such as HLA-A*01 and HLA- A*24 (40). In addition, it has been reported that approximately 90% of the epitopes presented by HLA-A*02:01 can also be presented by other subtypes of HLA-A*02 (41), suggesting that the identification of epitopes presented by the HLA-A*02 is of great interest for the therapeutic or prophylactic vaccine design in populations with a high frequency of this allele.

Regarding the GC9 epitope, which is found in the p17 protein, is located in a Gag region that presents high variability among the sequences belonging to the HIV-1 subtype B (42). In Los Alamos National Laboratory global HIV-1 sequence database, one of the variants reported for the GLLESSEGC (GC9) epitope is GLLESSEGC (S53T) (43), and CD8^+^ T-cells stimulated with S53T can produce IFN-γ (44). In genome alignments of strains belonging to epitopes of HIV-1 subtype B, a high frequency of S53T substitution is observed (43). Since this variant circulates in a high proportion of the studied population, it was considered the wild type variant of the GC9 epitope (GC9 WT) (15). Furthermore, Gesprasert et al. (45) reported that the amino acid variations at positions 5 and 6 of this epitope are directly caused by the HLA-A*02 allele. To the best of our knowledge, this is the first study that demonstrates a functional CD8^+^ T-cells response to the GLLETSEGC epitope and three mutated variants, two of them (S54A and S54T) associated with an increase in the HLA-I binding affinity.

Interestingly, we observed a higher quality of the CD8^+^ T-cell response to the S54T mutated variants, suggesting a relationship between HLA-I binding affinity and the antigen response. In the acute phase of infection, mutated peptide variants have low frequency and are thus poorly immunogenic; however, these mutated variants are relevant in the chronic phase of the infection, where a higher percentage of CD8^+^ T-cells are specific for mutated variants (46). Furthermore, viral variants in the chronic phase tend to be immunogenic, capable of inducing a CD8^+^ T-cell response characterized by cells expressing IFN-γ and strong cytotoxic response against CD4^+^ T-cells pulsed with the variants (46). Consistently, here it was observed that the S54A and S54T peptides induce a CD8^+^ T-cell response composed of IFN-γ, CD107a, and cytotoxic molecules. Besides, an association between the level of CD8^+^ T-cell polyfunctionality and the peptide-HLA-I binding affinity was observed; specifically, higher CD8^+^ T-cell polyfunctionality was found in response to the S54T peptide compared to S54A/E55G peptide, associated with lower HLA-I binding affinity. Some studies have shown that changes at the central position of an epitope may alter its recognition by the T cell receptor (TCR) (47, 48) and that the level of polyfunctionality is influenced by the affinity of the TCR-pMHC interaction (49). Thus, our findings suggest that position six is a relevant site for epitope recognition and that the change from a serine to a threonine may contribute to the avidity of the TCR-pMHC complex.

The SL9 epitope has been reported as immunodominant among chronically infected individuals expressing the HLA-A*02:01 allele. Approximately 75% of HLA-A*02^+^ individuals mount a CD8^+^ T-cell response against SL9 WT. Therefore, this epitope is an attractive candidate for immunotherapy (50). A study carried out by Boggiano et al. (51) showed that low affinity variants of SL9 impair the recognition of this epitope in HLA-A*02^+^ HIV-1-infected individuals, and therefore, the magnitude of the CD8^+^ T-cell response. However, the immune response induced by the mutated variant Y79F/T84V/L85F in the SL9 epitope has not been reported. A study by Jamieson et al. (52) observed that the Y79F/T84V mutated variant decreased by more than 50% the CD8^+^ T-cell response to SL9 WT, suggesting that the decrease in response may occur due to the loss of TCR recognition. In our study, there was a lower degranulation and *de novo* production of granzyme B, and lower polyfunctionality in response to Y79F/T84V/L85F compared to SL9 WT. Importantly, residues P8 and P9 located at the C-terminal region are critical anchor sites for the interaction of the peptide with the F binding pocket of the HLA-I molecule (53), so that mutations in these sites may alter the affinity of the pMHC complex and therefore, reduce the CD8^+^ T-cell response. Interestingly, a positive correlation between CD8^+^ T-cells producing *de novo* perforin and CD4^+^ T-cell counts was found in response to SL9 WT epitope, suggesting that an optimal response to SL9 epitope in these individuals could play a role in viral control. Indeed, perforin secretion by CD8^+^ T-cells plays a significant role in eliminating virus-infected cells is associated with the control of virus replication observed in long-term elite controllers/non-progressors (54).

Finally, consistent with our results, showing a functional competence of CD8^+^ T-cells in response to both polyclonal antigen-independent stimuli and HIV peptides, previous studies have observed that in HIV-1-infected individuals on prolonged cART, CD8^+^ T-cell dysfunction is partially improved, even though the levels of activation and exhaustion are maintained (55–57). Nevertheless, also, some studies indicate that the functional profile of HIV-1-specific CD8^+^ T-cells in treated individuals is maintained or even expanded (58). CD8^+^ T-cells from HIV-infected individuals improve their polyfunctional response to SEB after 24 weeks of cART. In contrast, this improvement is also observed for Gag-specific CD8^+^ T-cells after 114 weeks of therapy (55). Similarly, other studies have shown that IFN-γ^+^ HIV-specific CD8^+^ T-cells increase in magnitude after one year of suppressive cART (59). In line with these data, we observed a higher polyfunctional response to the mutated variants S54A and Y79F/T84V/L85F in individuals with longer treatment duration, suggesting that the quality of the CD8^+^ T-cells response to epitope variants may be maintained in the setting of low-level viremia induced by cART.

One of the main limitations of our study is that we did not sequence the circulating HIV strains in each individual to confirm the presence of the Gag-derived epitope variants. Furthermore, the emergence of mutations in HIV-1 strains is a stochastic process involving mutation rates (60) and the potential for the variation to persist (60). Thus, high and low affinity mutations might appear with the same probability, but these emerging mutations are selected depending on the virus replication fitness (61, 62) and the immune pressure (63). Nonetheless, our previous reports support the notion that a process of immune pressure most likely occurred in these individuals, providing a window of exposure to these peptides at some point of infection (15, 64). Another limitation is that this study considered only the HLA-I-peptide interaction; therefore, the TCR repertoire effect in recognizing the mutated peptides remains to be addressed, considering that changes in amino acids can affect also the molecular interaction with TCR (65) and that different clonotypes could constitute the Ag-specific T-cell population, which also may contribute to the quality response and the differences observed among individuals (66).

The present study characterized the CD8^+^ T-cells response to mutant Gag-derived epitopes of circulating strains in the HLA-A*02:01 context. Responses of CD8^+^ T-cells to the wild type and mutated epitopes were polyfunctional, producing multiple cytokines and cytotoxic molecules, resembling an effector profile. The key findings of this study are (i) Consistent with a chronic phase of HIV infection, we observed a high frequency of CD8^+^ T-cells that responded to the viral variants with the identified mutations; in most of the individuals, these variants have not displaced the response to the WT epitopes, and mainly induced a response mediated by IFN-γ^+^ and cytotoxic molecules expression. (ii) Quality of CD8^+^ T -cells responses (in terms of polyfunctionality) is associated with the binding affinity of the peptide to the HLA molecule. These results emphasize the importance of considering *in silico* HLA-I binding affinity predictions and evaluating the functional profile of CD8^+^ T cells to identify potential epitopes candidates for vaccine design.

## Data Availability Statement

The raw data supporting the conclusions of this article will be made available by the authors, without undue reservation.

## Ethics Statement

The studies involving human participants were reviewed and approved by Bioethics Committee from the Institute of Medical Research, School of Medicine, Universidad de Antioquia. The patients/participants provided their written informed consent to participate in this study.

## Author Contributions

AS-M performed the experiments and designed the figures. AS-M, LA-S, and PV analyzed the results and wrote the manuscript. JA-A, CA, FG, TR, SU-I, WC-M critically edited the manuscript. All authors contributed to the article and approved the submitted version.

## Funding

This study was supported by Universidad de Antioquia UdeA and COLCIENCIAS (111577757038; contract: 773-2017). AS-M was supported by Universidad de Antioquia UdeA, and Fundacion Sapiencia in Medellin.

## Conflict of Interest

The authors declare that the research was conducted in the absence of any commercial or financial relationships that could be construed as a potential conflict of interest.

## Publisher’s Note

All claims expressed in this article are solely those of the authors and do not necessarily represent those of their affiliated organizations, or those of the publisher, the editors and the reviewers. Any product that may be evaluated in this article, or claim that may be made by its manufacturer, is not guaranteed or endorsed by the publisher.

## Supplementary Material

Supplementary Figure 1 Gating strategy for the evaluation of the CD8^+^T-cell response. Representative dot plots showing the expression of cytokines, cytotoxic molecules and CD107a in unstimulated (negative control), Y79F/T84V/L85F peptide-stimulated (10 μg/ml), and SEB-stimulated cells.
