# Supplementary figures and images for: Functional Profile of CD8+ T-Cells in Response to HLA-A*02:01-Restricted Mutated Epitopes Derived from the Gag Protein of Circulating HIV-1 Strains from Medellín, Colombia
Source: Front Immunol. 2022 Mar 22;13:793982. doi: 10.3389/fimmu.2022.793982 (PMC8980466; doi:10.3389/fimmu.2022.793982)

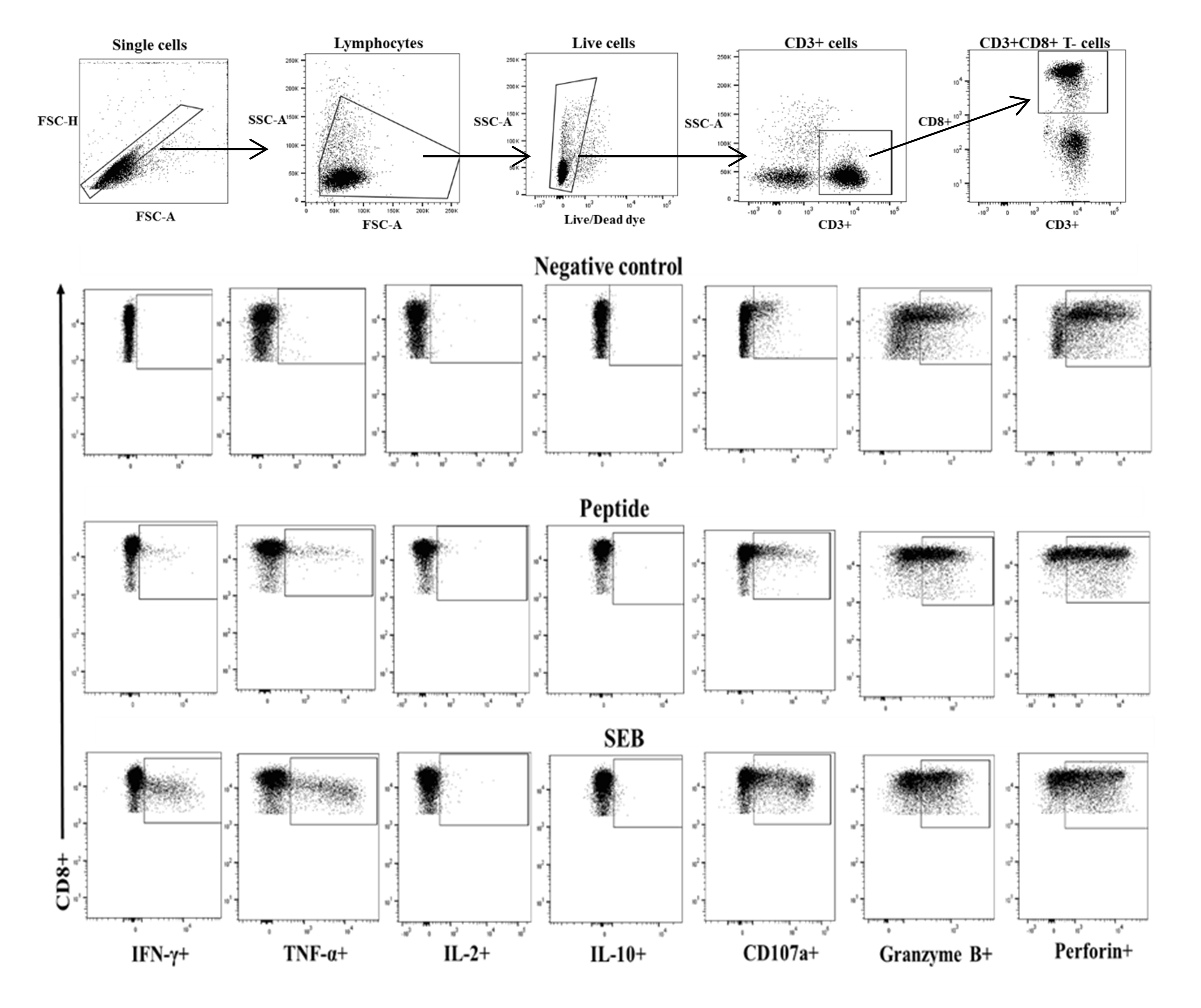

Supplement: Supplementary Figure 1 — Gating strategy for the evaluation of the CD8+T-cell response. Representative dot plots showing the expression of cytokines, cytotoxic molecules and CD107a in unstimulated (negative control), Y79F/T84V/L85F peptide-stimulated (10 μg/ml), and SEB-stimulated cells. [file Image_1.tif]
